# Angelica Sinensis Polysaccharide Prevents Hematopoietic Stem Cells Senescence in D-Galactose-Induced Aging Mouse Model

**DOI:** 10.1155/2017/3508907

**Published:** 2017-04-11

**Authors:** Xinyi Mu, Yanyan Zhang, Jing Li, Jieyu Xia, Xiongbin Chen, Pengwei Jing, Xiaoying Song, Lu Wang, Yaping Wang

**Affiliations:** ^1^Laboratory of Stem Cells and Tissue Engineering, Chongqing Medical University, 1 Yixueyuan Road, Yuzhong District, Chongqing 400016, China; ^2^Department of Histology and Embryology, Chongqing Medical University, 1 Yixueyuan Road, Yuzhong District, Chongqing 400016, China; ^3^Department of Pathophysiology, Chongqing Medical University, 1 Yixueyuan Road, Yuzhong District, Chongqing 400016, China

## Abstract

Age-related regression in hematopoietic stem/progenitor cells (HSC/HPCs) limits replenishment of the blood and immune system and hence contributes to hematopoietic diseases and declined immunity. In this study, we employed D-gal-induced aging mouse model and observed the antiaging effects of Angelica Sinensis Polysaccharide (ASP), a major active ingredient in dong quai (Chinese Angelica Sinensis), on the Sca-1^+^ HSC/HPCs in vivo. ASP treatment prevents HSC/HPCs senescence with decreased AGEs levels in the serum, reduced SA-*β*-Gal positive cells, and promoted CFU-Mix formation in the D-gal administrated mouse. We further found that multiple mechanisms were involved: (1) ASP treatment prevented oxidative damage as total antioxidant capacity was increased and levels of reactive oxygen species (ROS), 8-OHdG, and 4-HNE were declined, (2) ASP reduced the expression of *γ*-H2A.X which is a DNA double strand breaks (DSBs) marker and decreased the subsequent ectopic expressions of effectors in p16^Ink4a^-RB and p19^Arf^-p21^Cip1/Waf^ senescent pathways, and (3) ASP inhibited the excessive activation of Wnt/*β*-catenin signaling in aged HSC/HPCs, as the expressions of *β*-catenin, phospho-GSK-3*β*, and TCF-4 were decreased, and the cyto-nuclear translocation of *β*-catenin was inhibited. Moreover, compared with the positive control of Vitamin E, ASP exhibited a better antiaging effect and a weaker antioxidation ability, suggesting a novel protective role of ASP in the hematopoietic system.

## 1. Introduction

Hematopoietic pathophysiological changes like chronic anemia, decreased adaptive immune competence, and increased incidence of leukemia are closely associated with age [1]. As differentiation of tissue-specific stem and progenitor cells replaces worn out and damaged cells to maintain the normal homeostatic control, it has been proposed that the imbalances accompanying aging of the hematopoietic system may stem from reduction and/or dysfunction of hematopoietic stem/progenitor cells (HSC/HPCs) [1, 2]. HSC/HPCs with self-renewal and multilineage potential are the ancestor of all blood cells and lymphocytes. Exploring the possibility and the underlying mechanisms to delay HSC/HPCs senescence may provide promising ways to treat the age-related hematopoietic diseases and improve the quality of the senior life.

*Angelica Sinensis (olive) Diels *(dong quai) is a famous traditional Chinese medicine of treating hematologic and gynecological diseases for centuries. Angelica Sinensis Polysaccharide (ASP) is a major ingredient in Angelica Sinensis with significant bioactivities, such as antioxidant, antitumor, antiaging, antihepatotoxic, immunomodulatory, and neuroprotective effects [3]. Our recent studies have indicated an extraordinary antiaging role of ASP which protected HSC/HPCs against X-ray-irradiation-induced aging by inhibiting oxidative stress damage [4], alleviating DNA damage, and increasing telomerase activity [5]. But it still requires investigation in a more physiological-like aging model and further exploration of the molecular mechanisms.

Cellular senescence, a state of irreversible growth arrest, can be triggered by multiple mechanisms such as oxidative stress, telomere shortening, and DNA damage. D-galactose (D-gal) is a physiological reducing sugar that reacts with free amines of amino acids to form advanced glycation end products. Oversupply of D-gal generates oxidative stress by producing reactive oxygen species (ROS) through oxidative metabolism of D-gal as well as through glycation end products. Chronic systemic exposure of rodents to D-gal induces accelerated aging, including regression of bone marrow and the hematopoietic system [6, 7]. Thus, it was considered as an ideal model to study HSCs senescence.

In the recent decades, multiple molecules and the corresponding signaling pathways in stem cell senescence were discovered. The p16^INK4a^-Retinoblastoma (Rb) pathway and the p19^Arf^-Mdm2-p53-p21^Cip1/Waf1^ pathway, which can be induced by DNA damage response and regulate cell cycle arrest, are considered as important ways to regulate cellular senescence [8]. Moreover, Wnt/*β*-catenin signaling pathway is also involved in the regulation of stem cell senescence. The canonical Wnt signaling comprises a vast network of well-conserved proteins, which broadly influence embryogenesis and postnatal responses [9]. In the adult, Wnt signaling mediates the differentiation, maintenance, and proliferation of numerous stem cell types, including HSCs, and interacts with multiple signal transduction networks in aging process of stem cells, like TGF*β*, Hedgehog, and Notch signaling [10–13].

In the present study, we employed D-gal-induced aging mice model to further explore the antiaging role of ASP in HSC/HPCs in vivo. Our data have demonstrated that ASP had a significant antiaging effect via alleviating oxidative stress, preventing DNA damage, and inhibiting excessive activated of age-related signaling pathways. Generally, it enhanced the potential of ASP, the major active ingredient in Angelica Sinensis, to treat HSC/HPCs senescence and hematologic diseases.

## 2. Materials and Methods

### 2.1. Animal Treatment and Ethics Statement

Male C57BL/6J mice aged 6 to 8 weeks were purchased from the Medical and Laboratory Animal Center of Chongqing, China, and housed in a temperature- and light-controlled room with free access to water and food. All experiments were performed in accordance with institutional and national guidelines and regulations and were approved by the Chongqing Medical University Animal Care and Use Committee. All surgeries were performed under sodium pentobarbital anesthesia, and all the efforts were made to minimize suffering.

40 mice were randomly divided into 4 groups: D-gal group, mice were administrated with D-gal (120 mg/kg·bw), qd × 42 by subcutaneous injection; D-gal + ASP group, from 8th day of D-gal injection, the mice were given ASP (200 mg/kg·bw), qd × 35 at the same time by peritoneal injection; D-gal + VitE group (positive control), from 8th day of D-gal injecting, the mice were injected with Vitamin E (100 mg/kg·bw), qd × 35 at the same time by peritoneal injection; Control group, the mice were injected subcutaneously with saline at the same volume with D-gal for 42 days and peritoneally injected with saline at the same volume with ASP from the 8th day of the treatment.

### 2.2. Reagents

ASP (Purity ≥ 95%) was purchased from Ci Yuan Biotechnology Co. Ltd. Shaanxi (Xi'an, China) and dissolved in saline at the concentration of 20 mg/mL and sterilized by ultrafiltration. IMDM media: FBS were purchased from Gibco (CA, USA). The Anti-Sca-1^+^ Micro Bead kit was obtained from Miltenyi Biotech Co. (Bergisch Gladbach, Germany).

### 2.3. Collection of Sca-1^+^ HSC/HPCs

The mice were executed by cervical dislocation, and the femurs were collected. Bone marrow mononuclear cells (BMNC) were extracted from each group, and Sca-1 positive (Sca-1^+^) HSC/HPCs were separated by the magnetic activated cell sorting (MACS) technique as we previously described [14].

### 2.4. SA-*β*-Gal Cytochemical Staining

The Sca-1^+^ HSC/HPCs were collected after the treatment, and the senescence-associated *β*-galactosidase (SA-*β*-gal) staining was carried out according to the manufacturer's instructions (Beyotime Institute of Biotechnology, Shanghai, China). Briefly, 1 × 10^5^ purified cells were washed twice with PBS, fixed with 0.5% glutaraldehyde for 15 min at room temperature. Then, cells were washed with PBS and incubated with SA-*β*-Gal staining solution for 12 hours at 37°C without CO_2_ and light. Approximately 1 × 10^4^ cells were separated on each slide, and 400 cells were totally analyzed for each group. The percentages of SA-*β*-gal positive cells were calculated as counting the number of blue cells and dividing by the total number of cells.

### 2.5. CFU-Mix of Sca-1^+^ HSC/HPCs Cultures

After the treatment, Sca-1^+^ HSC/HPCs were collected. The mixed colony-forming unit (CFU-Mix) culture was performed as previously described [14]. Briefly, 4 × 10^5^ Sca-1^+^ HSC/HPCs were mixed with methylcellulose semisolid culture medium (Stem Cell Technologies, USA) and cultured in 24-well plates for 7 days in an incubator with 5% CO_2_ at 37°C. Then, the numbers of mixed colonies were counted in each group.

### 2.6. Measurement of ROS Levels

The Sca-1^+^ HSC/HPCs were collected after the treatment and reactive oxygen species (ROS) levels were analyzed by Reactive Oxygen Species Assay Kit (Beyotime Institute of Biotechnology, Shanghai, China) according to the manufacturer's instructions. In brief, 1 × 10^6^ Sca-1^+^ HSC/HPCs were collected in each group, washed by PBS, and incubated with fluorescent probes, 2′,7′-dichlorofluorescein diacetate (DCFH-DA), for 20 min with 5% CO_2_ at 37°C. Then, cells were washed by serum-free cell culture medium. Finally, ROS levels were detected by flow cytometry.

### 2.7. Detection of T-AOC Content

The Sca-1^+^ HSC/HPCs were collected after the treatment. The supernatant was obtained after sonication and centrifugation. Soluble protein concentrations were measured by the BCA procedure. The total antioxidant capacity (T-AOC) of the cells was detected by chemical colorimetric analysis of ferric reducing ability of plasma at 593 nm using enzymatic assay kit (Beyotime Institute of Biotechnology, Shanghai, China) according to the manufacturer's instructions.

### 2.8. Western Blotting Analysis

To detect *β*-catenin expression, the proteins in the cytoplasm and nucleus were extracted, respectively, from the Sca-1^+^ HSC/HPCs by Nuclear and Cytoplasmic Protein Extraction Kit (Beyotime Institute of Biotechnology, Shanghai, China) according to the manufacturer's instructions. Total proteins were extracted to detect the target proteins. Soluble protein concentrations were measured by the BCA procedure.

Samples containing 50 mg protein were separated on SDS-PAGE and transferred to PVDF membranes (Millipore, USA). Membranes were incubated overnight at 4°C with the primary antibodies against *β*-catenin, GSK-3*β*, Phospho-GSK-3*β*, TCF-4, *γ*-H2A.X (all purchased from Cell Signaling Technology, USA, catalog number: 9562, 9832, 9336, 2566, 2577), p16^INK4a^, Rb, p53, p21^Cip1/Waf1^, *β*-actin (all purchased from Santa Crus, USA, catalog number: sc-73434, sc-1538, sc-99, sc-397, sc-130656), and 4-HNE (ab46545, Abcam, USA), respectively. The HRP-conjugated secondary antibody was diluted at 1 : 5,000 in TBST. The membranes were visualized using the enhanced chemiluminescence detection system (Pierce, USA). The level of *β*-actin was used as an internal control. Relative intensities were quantified using Quantity One (Bio Rad).

### 2.9. Immunofluorescence Staining

Sca-1^+^ HSC/HPCs were gathered after the treatment and washed twice with PBS. The cell suspension was dispersed onto the glass slides. For immunofluorescence analysis, cells were fixed with 4% PFA for 10 min and washed with TBS with 0.3% Triton X-100 followed by blocking with 10% goat serum for 40 min at room temperature. Slides were then incubated with antibodies against *β*-catenin (1 : 100) (Cell Signaling Technology, USA) overnight at 4°C. Then, the cells were washed and incubated with Cy3-labeled secondary antibody (1 : 500, Beyotime Institute of Biotechnology, Shanghai, China) for 1 h at 37°C. PI was used for nuclear staining. All slides were viewed directly under a fluorescence microscope (LSM510; Carl Zeiss, Jena, Germany).

### 2.10. RNA Extraction and Real-Time Quantitative RT-PCR

The Sca-1^+^ HSC/HPCs were collected after the treatment. Total RNA was isolated using TRIzol reagent (Invitrogen, USA) according to the manufacturer's protocol. First-strand cDNA was created by Taqman RT reagents (Applied Biosystems, USA). Quantitative real-time PCR was performed using SYBR green Supermix (BioRad) on iCycler Real-Time Detection System (cfx96, BioRad). Expression levels of mRNA were normalized against *β*-*actin* and analyzed by the comparative cycle threshold method. The means ± SD of three independent experiments were determined. The PCR primers used are provided in the supporting information (Table S1 in File S1 Supplementary Material available online at https://doi.org/10.1155/2017/3508907).

### 2.11. Determination of 8-OHdG and AGEs Expression by ELISA

After the treatment, the serum in each group was collected, and the levels of 8-OHdG and AGEs were detected by ELISA kit (Shanghai Yuanye Bio-Technology, Shanghai, China) following the manufacturer's instructions. The concentrations were determined by comparing the OD values to the standard curves.

### 2.12. Statistical Analysis

Statistical analyses were performed using SPSS 19.0 software (SPSS, Inc., Chicago, IL, USA). Single factor ANOVA was used for comparison of mean values across the groups, and multiple comparisons were made by LSD test. *P* < 0.05 were considered significant.

## 3. Results

### 3.1. ASP Prevents Sca-1^+^ HSC/HPCs Senescence in D-Gal-Induced Aging Mice

The accumulation of advanced glycation end products (AGEs) in serum and tissues is an important biological indicator to evaluate the aging of the body [15]. After the treatment, we collected the serum of the mice and detected the AGEs concentrations by ELISA. In the D-gal group, content of AGEs was markedly higher than that of the control group. However, in the D-gal + ASP group, the AGEs concentrations were significantly attenuated compared to the D-gal group (Table 1).

**Figure 1:**
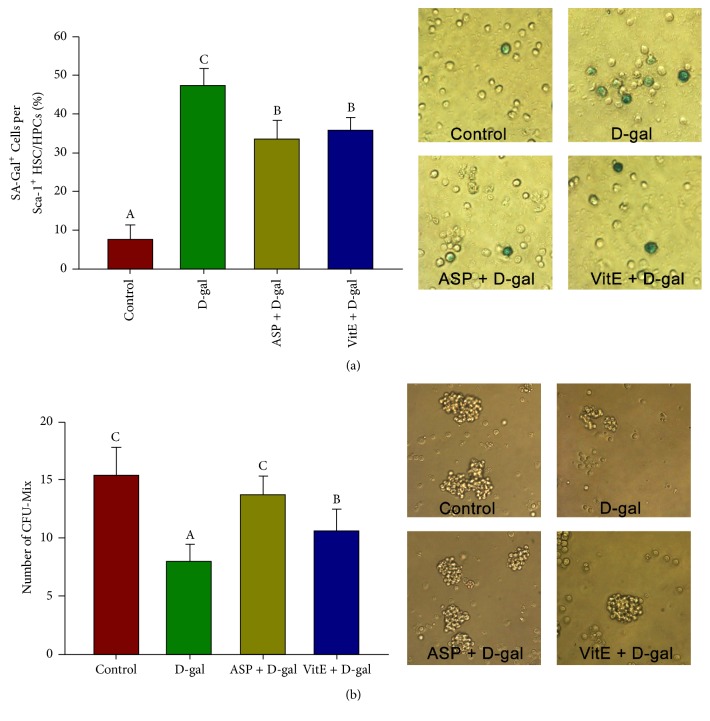
Effect of ASP on Sca-1^+^ HSC/HPCs senescence in D-gal-induced aging mice. The Sca-1+ HSC/HPCs were collected after the treatment. (a) The senescence-associated *β*-galactosidase (SA-*β*-gal) staining was carried out. The aged cells are stained in blue in the cytoplasm. Counting analysis of the stained cells was carried out. (b) The capacity of Sca-1+ HSC/HPCs to form hematopoietic progenitor colonies was evaluated by CFU-Mix culture. Counting analysis of the colonies was carried out. Different letters: *P* < 0.05.

SA-*β*-Gal staining is another classical biomarker for cell senescence assessment [6]. Aging cells are SA-*β*-Gal positive and stained in blue. Compared to the control, the ratio of SA-*β*-Gal positive Sca-1^+^ HSC/HPCs significantly increased in the D-gal group (47.4 ± 4.36% versus 7.6 ± 3.74%), while in the D-gal + ASP group, the ratio (33.6 ± 4.73%) was remarkably reduced compared to the D-gal group (Figure 1(a)).

The capacity to form CFU-Mix can reflect the potentials of HSC/HPCs of self-renewal and multipotential differentiation, and it decreases with the senescence of the HSC/HPCs [16]. Therefore, we also performed CFU-Mix cultures and counted the numbers of multipotential hematopoietic progenitor colonies. With consistence, the number of CFU-Mix in the D-gal group was significantly reduced than that of the control group (8.0 ± 1.43 versus 15.3 ± 2.51), but ASP recovered the CFU-Mix number in the D-gal administrated mice (13.7 ± 1.74) (Figure 1(b)).

All three analyses of senescence-associated tests showed that ASP significantly alleviated the D-gal-induced Sca-1^+^ HSC/HPCs senescence in vivo. Interestingly, compared to the D-gal + VitE group, in which the distinctive antioxidant Vitamin E was used as a positive control, the antiaging effect of ASP was equivalent or even more potent, especially in CFU-Mix formation (13.7 ± 1.74 versus 10.6 ± 1.86) (Figure 1(b)).

### 3.2. ASP Resists Oxidative Stress in Sca-1^+^ HSC/HPCs of D-Gal-Induced Aging Mice

Oxidative stress of ROS is one of the main causes of cellular senescence. The accumulation of ROS may cause oxidative damage to cell components including proteins, lipids, and nucleic acids, resulting in excessively produced nitrotyrosine, 4-hydroxynonenal (4-HNE), and 8-hydroxydeoxyguanosine (8-OHdG) [17]. Total antioxidant capacity (T-AOC) represents the enzymatic and nonenzymatic defense system of the body on free radical resistance. By evaluating ROS levels and also contents of 4-HNE, 8-OHdG, and T-AOC in the Sca-1^+^ HSC/HPCs we explored whether the antiaging effect of ASP was mediated by alleviating oxidative stress.

**Figure 2:**
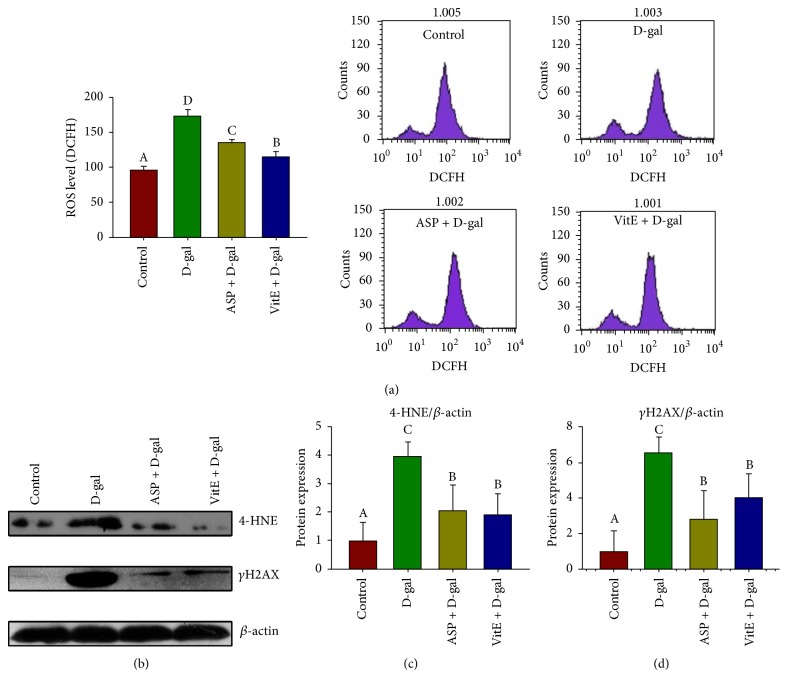
Effect of ASP on oxidative stress and DNA damage of Sca-1^+^ HSC/HPCs in D-gal-induced aging mice. The Sca-1+ HSC/HPCs were collected after the treatment. (a) The level of ROS was determined by DCFH fluorescence through flow cytometry. (b) The protein expression analyses of 4-HNE and *γ*H2A.X by Western blot, *β*-actin was used as the internal control. (c) The relative protein expression of 4-HNE. (d) The relative protein expression of *γ*H2A.X. Different letters: *P* < 0.05.

Compared with the control group, the cellular T-AOC content was declined (Table 2); meanwhile the cellular ROS levels and 4-HNE and 8-OHdG contents were elevated significantly in the D-gal group (Figure 2(a), Table 2). However, in the D-gal + ASP group, the cellular T-AOC content was elevated significantly, and the cellular ROS levels and 4-HNE and 8-OHdG contents were declined when compared with the D-gal group (Figure 2(a), Table 2). It demonstrated the potent antioxidative effect of ASP.

Unexpectedly, unlike its profound antiaging effect, ASP has less effect on oxidative stress than Vitamin E (Figure 2, Table 2). We speculate other mechanisms are involved in the antisenescence effect of ASP.

### 3.3. ASP Reduces DNA Damage in Sca-1^+^ HSC/HPCs of D-Gal-Induced Aging Mice

Cellular DNA damage accumulates with aging, triggering senescence. The phosphorylated H2A.X (*γ*-H2A.X) is considered as a sensitive indicator of DNA damage response (DDR) [18]. We detected *γ*-H2A.X expression of Sca-1^+^ HSC/HPCs by Western blot analysis to investigate the effect of ASP on DNA damage. It showed that the *γ*-H2A.X expression was increased in the D-gal group compared to the control group. However, ASP significantly decreased *γ*-H2A.X expression in the D-gal + ASP group, suggesting ASP reduced D-gal-induced DNA damage in Sca-1^+^ HSC/HPCs (Figure 2(b)). Moreover, compared to the D-gal + VitE group, this effect was more profound than that of Vitamin E, though not statistically significant.

### 3.4. Effect of ASP on p16^INK4a^-Rb and p19^Arf^-Mdm2-p53-p21^Cip1/Waf^ Signaling in Sca-1^+^ HSC/HPCs of D-Gal-Induced Aging Mice

**Figure 3:**
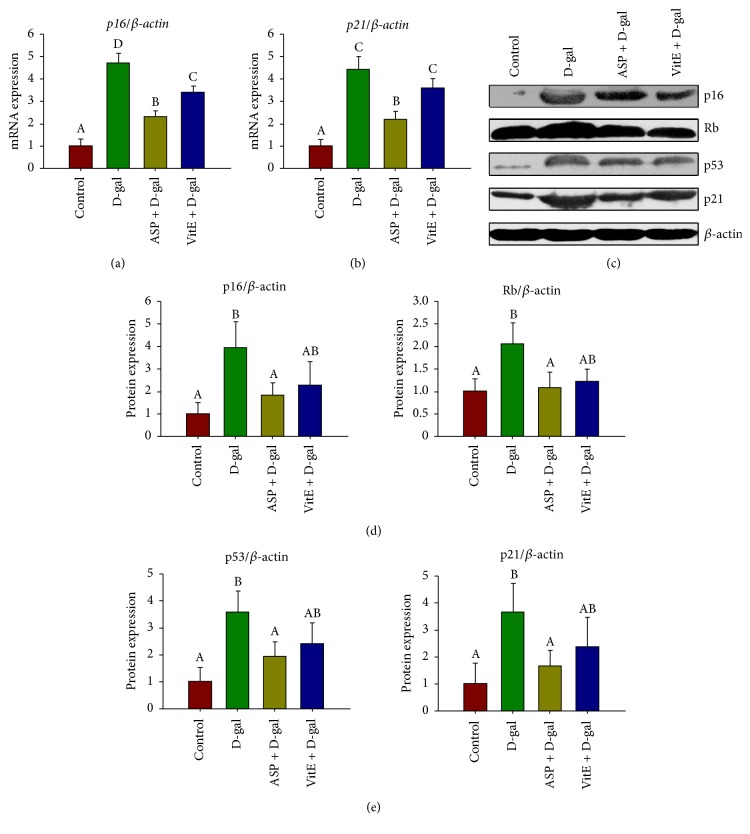
Effect of ASP on mRNA and protein expression of p16^INK4a^-Rb and p53-p21^Cip1/Waf1^ of Sca-1^+^ HSC/HPCs in D-gal-induced aging mice. The Sca-1+ HSC/HPCs were collected after the treatment. (a)* p16* mRNA expression analyses by qRT-PCR. (b)* p21* mRNA expression by qRT-PCR. (c) The protein expression analyses of p16^INK4a^, Rb, p53, and p21^Cip1/Waf1^ by Western blot, *β*-actin was used as the internal control. (d) The relative protein expression of p16^INK4a^-Rb signaling. (e) The relative protein expression of p53-p21^Cip1/Waf1^ signaling. Different letters: *P* < 0.05.

The p16^INK4a^-Rb pathway and the p19^Arf^-Mdm2-p53-p21^Cip1/Waf1^ pathway are considered as essential ways to regulate cell senescence [8]. We further explored whether the antisenescence effect of ASP was also mediated by the two pathways. By qRT-PCR analysis, it showed that mRNA expressions of* p16*^*INK4a*^ and* p21*^*Cip1/Waf1*^ were significantly increased in the D-gal group, comparing to the control; however, the mRNA expressions were reduced by ASP treatment (Figures 3(a) and 3(b)). It suggests a relationship between ASP and the two signaling pathways. We further confirmed the speculation by Western blotting analysis. It showed that the protein expressions of p16^INK4a^, Rb, p21^Cip1/Waf1^, and p53 were significantly higher in the D-gal group than those of the control group, while all were remarkably declined in ASP + D-gal group (Figures 3(c)–3(e)). Furthermore, compared to the D-gal + VitE group, this effect of ASP was more profound.

### 3.5. Effect of ASP on Wnt/*β*-Catenin Signaling Pathway in Sca-1^+^ HSC/HPCs of D-Gal-Induced Aging Mice

Studies have indicated that the excessive activation of Wnt/*β*-catenin signaling causes different degrees of stem cells senescence [19, 20]. The effect of ASP on Wnt/*β*-catenin signaling in HSC/HPCs is intriguing. We evaluated the expression of key signaling molecules of Wnt/*β*-catenin pathway to determine whether it was involved in the antiaging effects of ASP.

**Figure 4:**
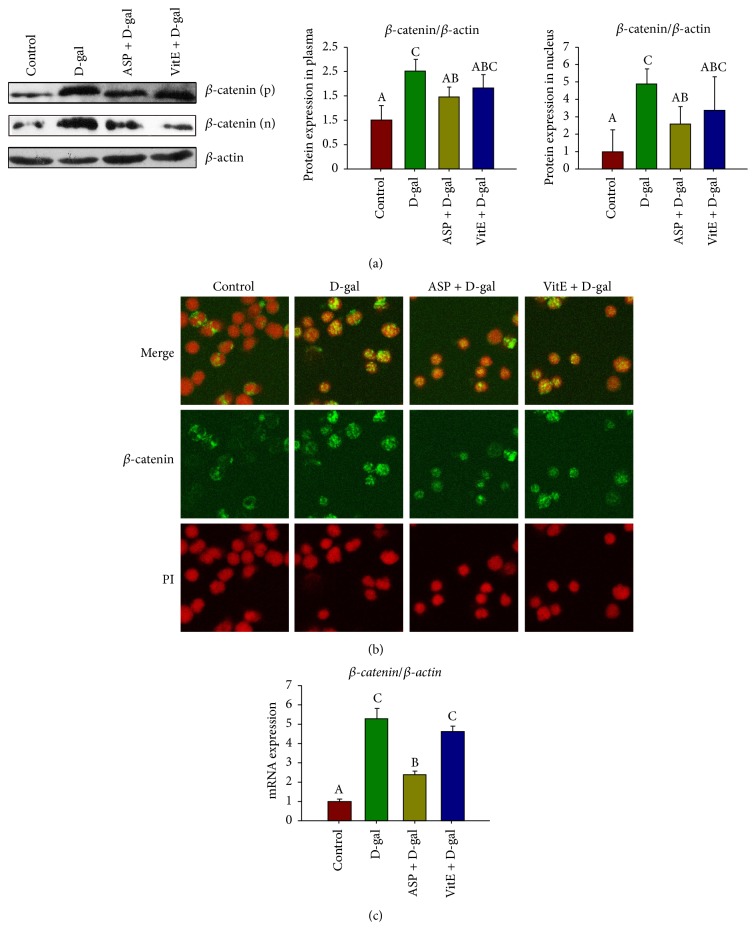
Effect of ASP on *β*-catenin expression of Sca-1^+^ HSC/HPCs in D-gal- induced aging mice. The Sca-1+ HSC/HPCs were collected after the treatment. (a) The protein expression analyses of *β*-catenin by Western blot. Proteins were extracted from cellular cytoplasm and nucleus, respectively; *β*-actin was used as the internal control. (b) The cellular localization of *β*-catenin by immunofluorescence, PI (red) to visualize nucleus. (c) *β*-*catenin* mRNA expression by qRT-PCR, *β*-actin was used as the internal control. Different letters: *P* < 0.05.

As a key signaling molecule, the level of *β*-catenin expression is a common index to detect the activation of canonical Wnt signaling pathway [21]. Besides, *β*-catenin per se has been shown to have a role in HSC/HPCs maintenance and self-renewal in murine model [12]. In repression of Wnt signaling, *β*-catenin is maintained at low cytoplasmic and nuclear levels, while *β*-catenin levels rise in cytoplasm and accumulate in the nucleus to activate downstream target genes expression during active Wnt signaling [10]. As qRT-PCR shown, the mRNA expression of *β*-*catenin* was upregulated in the D-gal group, compared to the control, and it was significantly attenuated in the ASP + D-gal group (Figure 4(c)). Furthermore, we extracted protein from cytoplasm and nucleus of Sca-1^+^ HSC/HPCs, respectively, and detected *β*-catenin expression by Western blotting analysis. As Figure 4(a) shown, the expression of *β*-catenin remarkably increased in both cytoplasmic and nuclear extracts in the D-gal group, compared to control group. However, in the D-gal + ASP group, *β*-catenin expression was significantly reduced compared with the D-gal group, especially in the nuclear extracts. We further confirmed the translocation of *β*-catenin by cytoimmunofluorescence with consistent results (Figure 4(b)).

**Figure 5:**
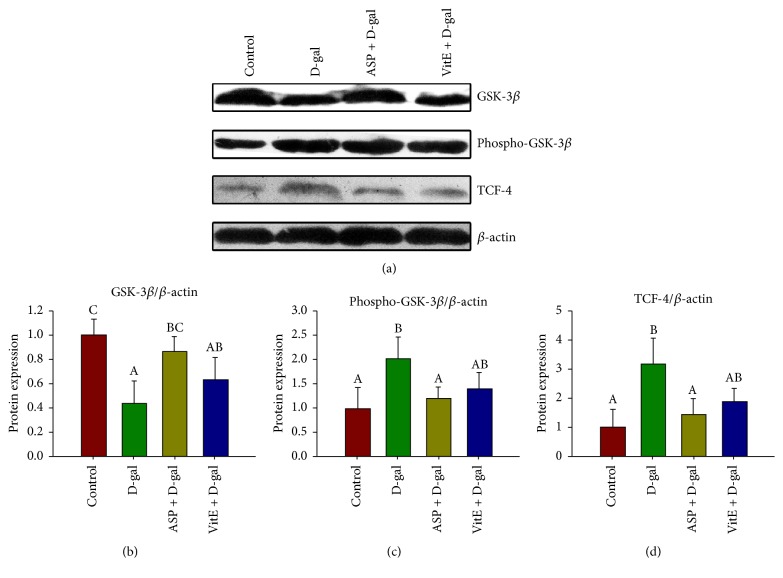
Effect of ASP on Wnt/*β*-catenin signaling mediators of Sca-1^+^ HSC/HPCs in D-gal- induced aging mice. The Sca-1+ HSC/HPCs were collected after the treatment. (a) GSK-3*β*, Ser9-phosphorylated GSK-3*β* and TCF-4 protein expressions by Western blot. *β*-actin was used as the internal control. (b) The relative protein expression of GSK-3*β*. (c) The relative protein expression of Ser9-phosphorylated GSK-3*β*. (d) The relative protein expression of TCF-4. Different letters: *P* < 0.05.

Glycogen synthase kinase-3*β* (GSK-3*β*) is an important negative regulator of Wnt/*β*-catenin signaling pathway, which is involved in degradation of *β*-catenin in the canonical pathway [10]. The inactivation of GSK-3*β* generally requires Ser9 phosphorylation [21]. We further explored GSK-3*β* and Ser9-phosphorylated GSK-3*β* (phospho-GSK-3*β*) expression in Sca-1^+^ HSC/HPCs by Western blot analysis. It showed that the GSK-3*β* expression was downregulated, and phospho-GSK-3*β* expression was upregulated in the D-gal group compared to the control groups. However, ASP treatment significantly reversed it in the ASP + D-gal group (Figures 5(a)–5(c)). It suggested that the GSK-3*β*-dependent degradation of *β*-catenin was enhanced by ASP treatment in the D-gal-induced aged Sca-1^+^ HSC/HPCs.

When Wnt/*β*-catenin signaling is activated, *β*-catenin translocates to nucleus, where it activates target gene expression through interactions with DNA-bound transcription factors, like T cell factor/Lymphoid enhancer factor (TCF/LEF) [22]. Therefore, we further detected TCF-4 expression by Western blotting analysis. As expected, TCF-4 expression was upregulated in the D-gal group compared to the control groups, while its ectopic expression was declined in the D-gal + ASP group (Figure 5(d)).

Collectively, these results suggest that excessive activation of Wnt/*β*-catenin/TCF-4 signaling is involved in the D-gal-induced senescence of Sca-1^+^ HSC/HPCs, and ASP treatment is able to inhibit this activation to resist stem cell senescence. Moreover, comparing with D-gal + VitE group, which represents the effect of antioxidant chemicals, ASP showed a more significant potential in this process (Figure 5).

## 4. Discussion

HSC/HPCs continuously replenish the cells that comprise the blood, including the immune system. Data from murine support an age-related decline in HSC function, suggesting that older HSCs are inadequate to cope with the demands of blood production and the immune system [23, 24]. Well-maintenance of the rejuvenating effect of stem cells provides approaches to delay senescence. Here, by the in vivo model of D-gal-induced aging mice, we found that ASP, a major active ingredient in Angelica Sinensis, exerts profound antiaging effect on Sca-1^+^ HSC/HPCs. Besides the potent antioxidant ability, ASP significantly affected multiple senescence-related signaling, including Wnt/*β*-catenin/TCF-4, p16^INK4a^-Rb, and p19^Arf^-Mdm2-p53-p21^Cip1/Waf1^, suggesting it as a promising molecule to delay senescence.

ASP is the initial extraction solvent of the roots of Angelica Sinensis (olive) Diels with water. It is a *β*-d-pyranoid polysaccharide with an average molecular weight of 72,900 Da. ASP has been proved to have beneficial effects in multiple disease models including cancer, leukemia, anemia, and inflammation [3, 25–27]. Among these effects, the antioxidant capability of ASP plays an important role. Specifically, ASP protects cultured neural cells from H_2_O_2_-induced cytotoxicity and reduces elevated intracellular ROS, but also prevents in vivo oxidative damage and ameliorates ischemic brain injury in rats [28]. It has also been showed that ASP attenuates concanavalin A-induced liver injury by decreasing ROS and increasing antioxidant enzyme activity [29].

Aging is a multifactorial process. Despite various hypotheses to explain the aging process, oxidative stress/free radical theory has been regarded as the main cause of aging in the literatures of past decades. The oxidative stress theory puts accumulation of ROS in the centre of processes that promote cell aging [30]. The highly reactive ROS are natural byproducts during energy production in mitochondria. Exogenously derived antioxidants and endogenous antioxidant enzymes degenerate ROS. An imbalance between ROS production and degradation results in oxidative stress, leading primarily to peroxidation of membrane fatty acid chains, modification of DNA, loss of sulfhydryl and carbonylation in proteins, and higher cell apoptosis rates [31, 32]. Oxidative stress ultimately causes cellular senescence and is closely correlated to life-history in vertebrates [33]. Specifically, it has been reported that aberrant increases in ROS can disrupt HSC quiescence by stimulating entry into the cell cycle, which comprises the ability of the HSCs to self-renew and leads to premature exhaustion [34–36]. Our previous study showed that superoxide damage was accumulating in the HSCs with the natural progress of aging [37].

D-gal administration model is a mimetic aging model related to free radical and the accumulation of waste substances in metabolism. D-Galactose (D-gal) is a physiological nutrient and a reducing sugar that reacts with free amines of amino acids in proteins to form advanced glycation end products through nonenzymatic glycation. As such, oversupply of D-gal could contribute to generation of ROS through oxidative metabolism of D-gal as well as through glycation end products [7]. Similarly, the accumulation of free radicals progressively damages the HSCs function during natural aging, while chronic systemic exposure of rodents to D-gal induces accelerated aging, including regression of bone marrow and the hematopoietic system [6, 7]. It is rational that the outstanding antioxidant capability of ASP can enhance the resistance of HSC/HPCs to D-gal-induced aging (Figure 1, Table 1, Figure 2(a), Table 2). In this study, we also employed Vitamin E, a common exogenous, diet-derived antioxidant, as an antioxidant positive control. Interestingly, though the antioxidant capability of ASP was not as great as Vitamin E (Figure 2(a), Table 2), ASP showed a more profound effect on antiaging (Figure 1, Table 1). It suggested a significant potential of ASP as an antiaging drug and other underlying mechanisms may be involved.

**Figure 6:**
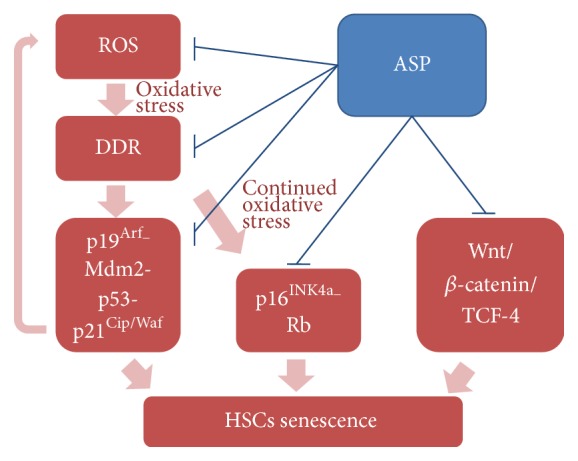
The summary of the effects of ASP on HSCs senescence.

In the process of aging, internal and external factors, including increasing oxidative stress and eroded telomeres, cause accumulation of cellular DNA damage and activation of DDR, which initiates and maintains the senescence growth arrest [8]. If the DNA damage exceeds a certain threshold, cells are destined to undergo either apoptosis or senescence [38]. Besides, DNA double strand breaks (DSBs) are especially potent senescence inducers [8]. The effect of ASP on expression of *γ*-H2A.X, the marker of DSBs, reinforced the antiaging role of ASP (Figure 2(b)). DDR subsequently triggers p16^INK4a^-Rb and p19^Arf^-Mdm2-p53-p21^Cip1/Waf^ pathways, which are two well-established senescent pathways engaging cell cycle regulation. ASP significantly alleviated the ectopic expression of the major components of the two pathways (Figure 3) which was consistent with its effect on DSB. However, senescence mediated by p16^INK4a^ and p21^Cip1/Waf^ activation can be uncoupled of DDR as well [8], leaving a possibility that the effects of ASP on DDR and the cell cycle engaged senescent signaling are independent. It is noteworthy that the two pathways may ultimately become engaged upon sustained senescence, because DNA damage initially halts cell cycle progression through p19^Arf^-Mdm2-p53-p21^Cip1/Waf^; if lesions persist, this activates p16Ink4a through p38-MAPK-mediated mitochondrial dysfunction and ROS production [39]. Therefore, it was rejoicing to find that the antiaging effect of ASP interrupted the recurrence at all stages (Figure 6).

As the mainstream hypothesis of aging causes, the oxidative stress theory of aging has recently been under debate, because experimental and correlative studies do not always support the hypothesis that high oxidative stress leads to shorter lifespan [40]. Emerging evidence indicates that alteration of signaling pathways caused by the change of stem cell microenvironment is one of the important causes of senescence [12]. Among them, the role of canonical Wnt/*β*-catenin signaling pathway in stem cell senescence was intriguing [19, 20]. The serum of aged mice can activate the Wnt/*β*-catenin signaling, and activation of *β*-catenin enforced cell cycle entry of HSCs, thus leading to exhaustion of the long-term stem cell pool. Furthermore, overexpression of *β*-catenin in adult mouse HSCs leads to impaired ability of multipotential differentiation, exhaustion of the pool of HSCs, and declined hemopoiesis [12, 41]. In the present study, ASP significantly attenuated excessive activation of Wnt/*β*-catenin/TCF-4 signaling in the D-gal-induced senescence of Sca-1^+^ HSC/HPCs. Besides, the phosphorylation of GSK-3*β*, which inactivates *β*-catenin degradation, was inhibited by ASP treatment (Figure 5). More importantly, as one of the major components of Wnt pathway, GSK3*β* is also a crosstalk integrator of multiple signal transduction networks. For example, GSK3*β* interacts with Notch signaling and PI3K/Akt signaling, which are all involved in stem cell aging [10]. In addition, studies suggest Wnt and Notch pathways belong to a network of regulatory circuits controlling the HSC pool [42]. Thus, besides the classic senescent theory of oxidative stress, the antiaging mechanism of ASP may be more extensive, and it requires further investigation.

Senescence of HSCs was closely linked to a variety of hematopoietic diseases. In the present study, we found an extraordinary antiaging effect of ASP on HSCs, and the effect was mediated by multiple mechanisms, including reducing oxidant stress and DNA damage and regulating senescence-related signaling pathways. Generally, ASP, a major constituent of initial extraction of the root, underlines the hematogenic and immunogenic element of Chinese Angelica Sinensis to some extent.

## Supplementary Material

The primers involved in quantitative real-time PCR were listed as below. For analysis of gene levels of interest, β-actin was used to normalise.

## Figures and Tables

**Table 1:** Effect of ASP on the level of AGEs in serum of aging model mice.

Groups	AGEs (pg/ml)
Control	320.7 ± 9.45^a^
D-gal	630.0 ± 15.5^c^
ASP + D-gal	588.7 ± 10.9^b^
VitE + D-gal	595.4 ± 11.05^b^

The contents of AGEs in serum were measured by ELISA kit. Data are expressed as mean ± SD (*n* = 10). Different letters: *P* < 0.05.

**Table 2:** Effect of ASP on the content of T-AOC and 8-OHDG in Sca-1^+^ HSC/HPCs of aging model mice.

Groups	T-AOC (U/mg protein)	8-OHDG (ng/ml serum)
Control	7.03 ± 0.63^a^	10.0 ± 1.02^a^
D-gal	3.53 ± 0.96^c^	40.42 ± 2.73^c^
ASP + D-gal	5.07 ± 2.06^b^	34.4 ± 2.36^b^
VitE + D-gal	4.87 ± 1.20^b^	32.4 ± 1.97^b^

T-AOC of the HSC/HPCs were detected by enzymatic assay kit. The contents of 8-OHDG in serum were measured by ELISA kit. Data are expressed as mean ± SD (*n* = 10). Different letters: *P* < 0.05.
