# Supplementary material for: Angelica Sinensis Polysaccharide Prevents Hematopoietic Stem Cells Senescence in D-Galactose-Induced Aging Mouse Model
Source: Stem Cells Int. 2017 Apr 11;2017:3508907. doi: 10.1155/2017/3508907 (PMC5405396; doi:10.1155/2017/3508907)
Supplement: Supplementary file 1 — The primers involved in quantitative real-time PCR were listed as below. For analysis of gene levels of interest, β-actin was used to normalise. [file 3508907.f1.docx]

**Supplemental Data**

**Materials and Methods**

Primers

| gene name | primer(5'-3') |
| --- | --- |
| *p16^INK4a^* | sense: CTCAGCCCGCCTTTTTCTTC antisense: CGCCTTCGCTCAGTTTCTCATG |
| *β-catenin* | sense: CGTGCGCATGGAGGAGATAGTAG antisense: CCCCTGCAGCTACTCTTTGGATA |
| *p21* | sense: TGCTCTTTTCCCCCACCCCATAC antisense: CCCCCACCACCACACACCATAGA |
| *β-actin:* | sense: GTGACGTTGACATCCGTAAAGA antisense: GCCGGACTCATCGTACTCC |
|  |  |
|  |  |
|  |  |
|  |  |
